# Changes in pain perception during pregnancy after one-time maximal physical exertion and an 8-week high-intensity interval training

**DOI:** 10.3389/fphys.2023.1304534

**Published:** 2023-12-12

**Authors:** Katarzyna Leźnicka, Agata Gasiorowska, Maciej Pawlak, Aleksandra Jażdżewska, Agnieszka Maciejewska-Skrendo, Monika Chudecka, Anna Szumilewicz

**Affiliations:** ^1^ Faculty of Physical Culture, Gdansk University of Physical Education and Sport, Gdansk, Poland; ^2^ Faculty of Psychology in Wroclaw, SWPS University of Social Sciences and Humanities, Wroclaw, Poland; ^3^ Department of Physiology and Biochemistry, Poznan University of Physical Education, Poznan, Poland; ^4^ Institute of Physical Culture Sciences University of Szczecin, Szczecin, Poland

**Keywords:** pregnancy, high-intensity interval training, pain tolerance, pain threshold, cardiopulmonary exercise test

## Abstract

**Background:** Pain, a subjective sensation, poses a great challenge to the human body as a stressor. There is empirical evidence that moderate to intense physical activity increases pain tolerance and this may be critical during pregnancy for optimal pregnancy, fetal development, and delivery. To the best of our knowledge, it is the first study examining the changes in pain perception in pregnant women after a maximal progressive exercise test and after 8 weeks of high-intensity interval training (HIIT).

**Methods:** Thirty-five women with uncomplicated singleton pregnancies between 13 and 28 weeks of gestation participated in the study. The HIIT intervention was developed in accordance with the recommendations and available data on HIIT during pregnancy. The maximal progressive cardiopulmonary exercise test was performed on a cycle ergometer with an electronically controlled load. Pressure pain threshold and pressure pain tolerance were measured with an algometer.

**Results:** We found significant effects of the maximal exercise test and high-intensity interval training, such that the pregnant women had higher pain tolerance after the maximal exercise test than before and after the high-intensity interval training than the baseline.

**Conclusion:** Our results suggest that post-exercise analgesia may be important in pregnant women and that high-intensity interval training appears to be beneficial for pregnant women to improve their pain tolerance while being obstetrically safe. Increased pain tolerance before labor could lead to better management of pain during labor and in the postpartum and lactation periods. Increasing pregnant women’s awareness of this issue can improve their wellbeing and provide more comfort during labor.

## 1 Introduction

Pain, a subjective sensation, poses a great challenge to the human body as a stressor. A notable factor contributing to the variability of human pain perception is the interplay of genetic and environmental influences (Leźnicka et al., 2018), as well as psychological, social, cultural, and spiritual elements. In addition, many variables such as location and duration of pain, patient-specific characteristics, especially personality or temperament, previous pain experiences, life satisfaction, social relationships, and physical activity (PA) lead to a unique and individualized shaping of the intensity and quality of pain, as well as a personal relationship with the experience of pain. These factors determine whether a particular noxious stimulus is perceived as more or less painful (Pawlak et al., 2019). Regular physical activity, especially in contact sports such as team games and combat sports enforces pain tolerance.

The complex origin of pain may explain the beneficial effects of regular physical activity (PA), which translates into increased pain tolerance (Koltyn, 2000). Physically active people are better able to cope with stress, which enables them to increase their PA level and ultimately leads to less pain (Koltyn, 2002). Most studies on the relationship between pain perception and PA refer to young, physically active people. However, there are no reports on the effects of PA in pregnant women, for whom such additional stimuli may be perceived differently than in non-pregnant women. Furthermore, even low levels of physical exertion have been shown to alter the perceived threshold for painful and non-painful stimuli (Pawlak, 2013). This finding is supported by a meta-analysis (Tesarz et al., 2012) and a separate study on martial arts (Leźnicka et al., 2016) demonstrating higher pain tolerance in athletes than in nonathletes. These authors suggest that regular PA is associated with altered pain perception, although the effects on pain threshold are still unclear. This phenomenon, termed “acute exercise-induced hypoalgesia,” is usually of short duration, lasting less than 30 min after a single exercise session (Tesarz et al., 2012). In addition, high levels of physical activity have been associated with increased conditioned pain tolerance in healthy individuals (Lemley et al., 2015), so the effects of PA may be particularly beneficial during childbirth, and act as “acute exercise-induced hypoalgesia” (Brown, 2002; Hinman et al., 2015; Morales-Suárez-Varela et al., 2021).

PA has been recommended for years as an essential component in promoting a healthy course of pregnancy (WHO, 2020a). Pregnant women who exercise regularly have fewer complications, injuries, musculoskeletal trauma, and maternal harm during delivery (Davenport et al., 2019). The beneficial effects of PA have also been demonstrated in animal model studies of pregnant mice (Parent Vachon et al., 2019). Despite these conclusions, the literature lacks meaningful studies on the impact of physical activity on pain perception during pregnancy, labor, and the postpartum period, as well as research examining changes in pain perception during pregnancy. Most studies have focused exclusively on changes in the subjective perception of persistent pain, relying solely on self-reports. Other studies have examined pain perception in response to mechanical stimulation or cold using the Cold Pressor Test (CPT) (Ohel et al., 2007; Skovbjerg et al., 2016). However, most of these studies have focused primarily on analyzing specific factors that affect only postpartum pain, making it difficult to extrapolate their findings to general pain sensitivity (Berlit et al., 2018). In this project, we aim to close this gap by 1) using objective measures of pain sensitivity (PPT and PTOL) instead of self-reports and 2) investigating how this pain sensitivity is influenced by physical training during pregnancy.

In recent years HIIT has become a training method that improves fat-burning potential and anaerobic threshold (AT) in both healthy individuals and patients. Positive effects of HIIT interventions have been observed in various populations including clinical populations (Campbell et al., 2019; Lavin Perez et al., 2021). Interestingly, results of HIIT interventions concerning the improvement of reproductive functions have been observed in women (Kiel et al., 2020) and men (Hajizadeh et al., 2020) with infertility. Given the superiority of HIIT programs over moderate-intensity continuous training (MICT) in various study groups, both in terms of benefits in metabolic changes and in affecting the psychological wellbeing of participants, pregnant women are poised to continue or begin participation in HIIT programs. Performing HIIT during pregnancy is safe in terms of obstetric outcomes and well tolerated by pregnant participants, while providing them with the enjoyment of exercise. HIIT interventions either led to an improvement in selected maternal and fetal health parameters or had no impact. No adverse effects were observed (Szumilewicz et al., 2022b) Nevertheless, to date, there are no official guidelines for programming and implementing HIIT programs during pregnancy.

In 1990s, there was a widespread belief that pregnant women should avoid anaerobic exercise such as sprints or interval training (Ohel et al., 2007). Given subsequent evidence from human populations of the beneficial effects of prenatal exercise on maternal and fetal health, the question is no longer “if” but “how” pregnant women should exercise (Lox and Darrena, 2000). It also raises a question of how and what exercise goals they can achieve by participating in prenatal HIIT programs. Systematic reviews on this topic have found that pregnant women can benefit from HIIT in terms of improving fitness and health parameters of both the mother and newborn. However, there are no data on how HIIT affects pain perception during pregnancy. Moreover, given the mechanism of “acute exercise-induced hypoalgesia” it can be hypothesized that high or maximal intensity exercise would also have an impact on pain perception during pregnancy. Therefore, in the current study we examined changes in pain perception in pregnant women, hypothesizing that 1) participation in a maximal progressive cardiopulmonary exercise test (CPET) and 2) participation in an 8-week high-intensity interval training (HIIT) would increase objective pain tolerance in pregnant women.

## 2 Materials and methods

### 2.1 Participants

46 participants volunteered to participate in the 8-week HIIT program. As 11 of them did not fulfill the eligibility criteria, they were not qualified to the study. The eligibility criteria were: 1) normal course of pregnancy confirmed during the standard obstetric assessment following Polish national law; 2) consent of the obstetric care provider to participate in the study tests and exercise classes; 3) week of gestation not higher than 28 to be able to attend the entire intervention before giving birth; 4) age between 18 and 45 years old; 5) availability to participate in classes three times a week during the 8-week exercise programme; The exclusion criteria were as follows: 1) contraindications to increased physical exertion or health conditions that could adversely affect the safety of the participants or fetuses or the quality of the gathered data; 2) multiple pregnancy; 3) lack of access to internet and MS Teams software.

As a result, thirty-five pregnant women in singleton, uncomplicated pregnancy entered the 8-week HIIT program (aged 24–42; *M* = 31.26, *SD* = 4.22; week of pregnancy 13–28, *M* = 20.31, *SD* = 4.23). Half of them (*n* = 18, 51.43%) did not give birth before. One woman did not complete the program and did not participate in the second pain sensitivity measurement. The baseline characteristics of the study sample at the beginning of the intervention are presented in Table 1.

**TABLE 1:** Baseline characteristics of the study group.

Characteristics	Min	Max	*M*	Mdn	*SD*
Age	24	42	31.26	30	4.22
Week of pregnancy	13	28	20.21	20	4.23
BMI	19.6	30.9	24.52	24.20	2.75
Number of previous childbirths	0	5	0.91	0	1.31
Dominant limb	94.29% right, 5.71% left				
Previous childbirth	51.43% no, 48.57% yes				
IPAQ category	17.14% low, 45.71% medium, 37.14% high				

Min–minimum; Max–maximum; M–mean; Mdn–median; SD, standard deviation.

### 2.2 Procedure

During the recruitment phase, research-relevant personal data were collected, and informed consent was obtained from study participants. Then, the short version of the International Physical Activity Questionnaire (IPAQ) (Cheng, 2016) was used to determine the level of physical activity before the intervention. Participants were then invited to the physical effort laboratory to measure pain sensitivity. Objective pain perception, operationalized as pain pressure threshold (PPT) and pain tolerance (PTOL), was measured with an algometer. Subjective pain perception was performed with the Visual Analog Scale (VAS) used to measure clinical pain. After these assessments, participants completed a maximal progressive cardiopulmonary exercise test (CPET) followed by a 3-min rest period. After the rest period, PPT, PTOL, and subjective pain assessments were performed again.

Upon completing the 8-week HIIT program, participants were tested again to measure postintervention pain sensitivity, following the same protocol as at the baseline. Measurements of PPT, PTOL, and subjective pain sensation were obtained, followed by CPET with a 3-min rest interval. After the rest interval, a final measurement of PPT, PTOL, and subjective pain was performed.

### 2.3 HIIT training

The HIIT intervention was developed based on analysis of recommendations for the design and implementation of prenatal exercise programs (Szumilewicz and Santos-Rocha, 2002; Santos-Rocha et al., 2022) and a review of available data on HIIT during pregnancy (Szumilewicz et al., 2022a). Online classes were held three times a week for 8 weeks via MS Teams. They were led by exercise specialists trained according to the European Lifelong Learning Standards “Exercise in Pregnancy and Postpartum” (Szumilewicz et al., 2022b). Each session included 7–10 min of warm-up, training, and instructions on how to perform the exercises in the main part, how to breath properly, how to activate the pelvic floor muscles, and how to keep the correct posture.

The main part of the session consisted of high-intensity intervals lasting 15–20 min. The ratio of exercises to rest was set at 1:2, 1:1, or 2:1, depending on the participant’s abilities, stage of pregnancy, and progress of the training program. The duration of each exercise ranged from 30 to 45 s (Table 2). Each session, regardless of the week of the program, consisted of four sets (workout + rest breaks) and four cycles (exercises).

**TABLE 2:** The characteristics of HIIT intervention.

Week number	Time of workout interval (s)	Time of rest interval (s)	Time between sets (s)
1	30	60	60
2	30	60	30
3	45	45	60
4	45	45	45
5	45	45	30
6	30	30	60
7	30	30	30
8	30	15	30

During the exercise intervals, participants were expected to exceed the anaerobic threshold intensity (AT), determined individually based on CPET results. The AT was determined utilizing a modified V-slope method and the ventilatory equivalent (VE) method (Beaver et al., 1986). To monitor exercise intensity, all participants used heart rate monitors, the 0–10 Borg Rating of Perceived Exertion (RPE) Scale (Borg, 1998) and the Talk Test (Persinger et al., 2004). After the interval part of the session, participants performed resistance, postural, neuromotor, and stretching exercises for 5–10 min. The cool-down consisted of birthing preparation and pelvic floor muscle exercises, as well as relaxation and visualization of pregnancy and labor (up to 20 min). The women did not use any exercise equipment, but only the resistance of their body weight. We tailored this HIIT intervention to the needs and abilities of the pregnant women based on diagnostic and functional tests (e.g., related to pelvic floor muscle function, possible back pain, and pregnancy discomfort) performed before the intervention. The program was offered to pregnant women regardless of their fitness level or motor skills. We did not observe any adverse effects of our HIIT intervention on pregnancy development, delivery, or neonatal. conditions.

### 2.4 Pain measurement

Pain pressure threshold (PPT) and pressure pain tolerance (PTOL) were assessed using an algometer from Medoc AlgoMed (Israel). PPT is defined as the minimum pressure necessary for the sensation of pressure to first change to pain. PTOL describes the maximum stimulus intensity or duration of continuous painful stimulation that a person is willing to endure.

Before testing, participants were informed about the functioning of the device and then a test measurement was performed. Measurements were taken in a sitting position on the dorsum of the hand between the thumb and index finger, and on the lateral surface of the arm at a distance of 2/3 of the shaft length. All measurements were taken in the morning. The experimenter placed the algometer head on the area under examination and applied stimuli uniformly at a rate of 30 kPa/sec. When the participant felt pain, she was instructed to say ‘stop.’ This measurement was scored as the pain threshold (PPT). The measurement continued until the participant could no longer tolerate the stimulus, at which point they indicated the end of the measurement. The point at which a painful pressure stimulus could no longer be endured was documented as the pain tolerance measurement (PTOL).

After completing the PPT and PTOL assessments, participants were asked to provide self-reported pain intensity using the Visual Analog Scale (VAS) to assess the extent of subjective pain during the procedure compared with their personal perception of pain. Pain intensity was rated on a scale from 0 = “complete absence of pain and discomfort” to 10 = “experiencing the most severe possible pain and discomfort.” We used the VAS as a measure of pain perception in addition to objective pain metrics (PPT, PTOL) to compare objective and subjective aspects of pain sensitivity in response to physical activity.

### 2.5 The cardiopulmonary exercise test (CPET)

The maximal progressive cardiopulmonary exercise test (CPET) was performed in accordance with the American Thoracic Society/American College of Chest Physicians guidelines (Galluci, 2019) on a cycle ergometer with electronically controlled load (Viasprint 150P; Bitz, Germany).

Participants began the protocol by cycling for 4 min at a relative load of 0.4 W·kg^−1^ of body weight to allow for an adequate warm-up. After the warm-up, the load was continuously increased by 0.2 W·kg^−1^ per minute until participants reached their maximum capacity. Participants were motivated to exert themselves to their limits and were informed they could cease the test anytime. Due to the specificity of the group that participated in our study, the participants were instructed to perform the effort “as much as possible”, but whenever they felt unwell, they could interrupt the test. After cycling, participants were given a recovery period of 3 min.

### 2.6 Ethics statement

The present research was approved by the Bioethics Committee of the Regional Medical Chamber in Gdansk (Poland), number KB-8/21. The research protocols adhered to the ethical principles of the Declaration of Helsinki of the World Medical Association.

Before participating in the study, participants were fully informed about its aims and gave their written consent to participate. Anonymity was ensured for all personal data and results, and they were processed and stored in accordance with applicable data protection regulations in Poland. This study is an integral component of a clinical trial registered at ClinicalTrials.gov (NCT05009433).

### 2.7 Statistical analysis

The data analysis was performed with JAMOVI (Galluci, 2023; Goss Sampson, 2022). The threshold for statistical significance was set at *p* < 0.05. The data were considered nested, as all participants were subject to pain measurements several times. Hence, multi-level modeling with JAMOVI with REML estimation, allowing for using variables that deviate from the normal distribution, was applied for data analysis.

The mixed-model regressions included the independent variables indicating whether the pain-related variables were measured: 1) before or after HIIT intervention, 2) before or after the CPET test; 3) on dominant vs. non-dominant limb; 4) on hand vs. arm. The regression also included the interactions between the abovementioned variables and included a random intercept for participants. The regression analysis was conducted three times for the following dependent variables: 1) pain threshold, 2) pain tolerance, and 3) subjective feeling of pain. All predictors were effect-coded, and all dependent variables were z-scored before the analysis to allow for standardized coefficients.

The sensitivity analysis using G*Power (Faul et al., 2007) revealed that with power 1—*β* = 80% and significance *α* = 0.05, a sample of *n* = 35 and 16 measurements from each participant correlated on average at .40 is large enough to detect a main effect of *β* = 0.13, a two-way interaction of *β* = 0.26, and a three-way interaction of *β* = 0.36 in within-group comparisons.

## 3 Results

### 3.1 Pain threshold

The results of multi-level regression conducted with JAMOVI (Table 3) showed that the independent variables and covariates accounted for R^2^_marginal_ = 3.30% of the variance in PPT. The effect of measurement on hand vs. arm was significant, such as the pain threshold was higher when measured on the hand than on the arm. The effect of measurement on the dominant vs. non-dominant limb was significant, with a higher pain threshold on the latter than on the former. The effect of CPET was only marginally significant, with the pain threshold being lower after the exercise test than before. Most importantly, the effect of HIIT intervention was not significant. We also found no significant interactions between the HIIT intervention, CPET test, and place of pain measurement. In sum, the effect we found was weak and unrelated to physical activity intervention.

**TABLE 3:** The results of the multilevel regression analyses for PPT, PTOL, and VAS scores. Fixed effects parameters estimates.

	PPT				PTOL				VAS			
Predictor	β	SE	t	p	β	SE	t	p	β	SE	t	p
hand	**.16**	**.08**	**2.03**	**.043**	**.43**	**.06**	**7.01**	**.001**	**.12**	**.06**	**2.10**	**.036**
HIIT	−.11	.08	−1.41	.159	**.43**	**.06**	**7.09**	**.001**	**.31**	**.06**	**5.22**	**.001**
CPET	−.15	.08	−1.88	.061	**.16**	**.06**	**2.55**	**.011**	−.03	.06	−0.52	.600
dominant	**−.18**	**.08**	**−2.19**	**.029**	**.16**	**.06**	**2.59**	**.010**	**−.25**	**.06**	**−4.17**	**.001**
hand ✻ HIIT	.24	.16	1.49	.138	.08	.12	0.67	.503	−.14	.12	−1.16	.247
hand ✻ CPET	−.24	.16	−1.47	.142	−.01	.12	−0.11	.910	−.05	.12	−0.45	.651
HIIT ✻ CPET	−.16	.16	−1.02	.307	.13	.12	1.05	.296	.12	.12	0.99	.321
hand1 ✻ dominant	−.07	.16	−0.43	.669	.03	.12	0.23	.816	.00	.12	0.01	.994
HIIT ✻ dominant	−.08	.16	−0.50	.615	−.07	.12	−0.55	.581	.12	.12	1.04	.299
CPET ✻ dominant	−.05	.16	−0.28	.776	.01	.12	0.12	.904	**.28**	**.12**	**2.37**	**.018**
hand ✻ HIIT ✻ CPET	−.11	.32	−0.35	.729	−.15	.24	−0.62	.537	−.34	.23	−1.43	.154
hand ✻ HIIT ✻ dominant	.13	.32	0.40	.687	−.03	.24	−0.11	.916	.11	.23	0.46	.644
hand ✻ CPET ✻ dominant	−.13	.32	−0.42	.678	.00	.24	0.02	.986	.05	.23	0.23	.820
HIIT ✻ CPET ✻ dominant	.17	.32	0.54	.589	−.05	.24	−0.22	.828	−.04	.23	−0.18	.859
hand1 ✻ HIIT ✻ CPET ✻ dominant	.31	.64	0.49	.625	−.41	.49	−0.84	.400	−.18	.47	−0.38	.701

Note: Satterthwaite method for degrees of freedom. PPT, pain pressure threshold; PTOL, pain tolerance; VAS, subjective pain measured with Visual Analog Scale; HIIT, 8-week High-Intensity Interval Training (after vs. before); CPET, maximal progressive cardiopulmonary exercise test (after vs. before); dominant = measurement on dominant vs. nondominant limb; hand = measurement on hand vs. arm. Values in bold represent statistically significant effects at *p* < 0.05.

### 3.2 Pain tolerance

The same analysis with pain tolerance as a dependent variable (Table 3) demonstrated that the independent variables and covariates accounted for R^2^_marginal_ = 10.57% of the variance in PTOL. The effects of PTOL measurement on the dominant vs. non-dominant limb were significant (Table 3), such that pain tolerance was higher when measured on the dominant limb than on the non-dominant limb. Also, the effects of PTOL measurement on hand vs. arm were significant, such as pain tolerance was higher when measured on the hand than when measured on the arm.

Most importantly, we found significant effects of the maximal exercise test and HIIT intervention, such that participants demonstrated higher pain tolerance after the CPET than before and after the HIIT intervention than at the baseline. We also found no significant interactions between the HIIT intervention, CPET, and place of pain measurement, meaning that the effects of acute analgesia and the 8-week training program are independent and universal concerning where the pain tolerance was measured. In sum, we found a moderate overall effect of our interventions on pain tolerance, with the effect of HIIT being much stronger than the effect of CPET.

### 3.3 Subjective pain (VAS score)

Regarding subjective pain perception, regression analysis showed that the independent variables and covariates accounted for R^2^_marginal_ = 5.22% of the variance in the dependent variable (Table 2). A significant effect was observed for the dominant vs. non-dominant limb, such that subjectively, the experience of pain was weaker in the dominant limb than in the nondominant limb. We also found a significant effect of the hand vs. arm, such that the experience of pain was stronger on the former than on the latter. Most importantly, we found a significant effect of HIIT intervention, such as the subjective experience of pain was stronger after completion of the training program than at the baseline. The effect of the CPET was not significant. However, the only significant interaction we found was between the CPET and measurement on the dominant vs. non-dominant hand. Further decomposition of this interaction revealed that the subjective experience of pain on the non-dominant hand was weaker after the CPET than before it, β = −.17, se = .08, *t* = −2.05, *p* = .041. However, the CPET did not affect the subjective feeling of pain on the dominant hand, β = .11, se = .08, *t* = 1.31, *p* = .192. In sum, we found a weak effect of our interventions on subjective pain perception, with HIIT as the only meaningful factor.

## 4 Discussion

To our knowledge, this is the first study investigating the changes in pain perception in pregnant women after a maximal progressive exercise test and after 8 weeks of high-intensity interval training. Athletic fitness training, especially high-intensity interval training (HIIT; short, intense workouts with rest or active recovery), has attracted increasing interest from researchers worldwide in recent years (Feito et al., 2018). This type of training not only improves cardiovascular performance, but also leads to a beneficial reorganization of cellular structures, increases skeletal muscle mitochondrial activity, modulates glucose and lipid metabolism, and decreases pain sensitivity (Wu et al., 2021).

The World Health Organization and experts in the field of gynecology recommend at least 150 min per week of moderate-to high-intensity exercise during pregnancy unless contraindications exist (Mottola et al., 2018; WHO, 2020b; ACOG, 2021). It appears that high-intensity interval training (HIIT) may be a good exercise option for pregnant women. Among its many benefits such as improving fat burning potential and AT in both healthy individuals and patients, including those with cardiovascular disease (Wisløff et al., 2007), cancer (Devin et al., 2016), or obesity (Buckinx et al., 2018), it is also beneficial in the management of chronic pain. HIIT can be an important adjunct to conventional drug therapies and improve the quality of life of various populations (Botta et al., 2022). It should be noted that most studies published to date have focused on older women who have already passed menopause or on elite athletes. There are no data yet describing the effect of HIIT modulating pain in pregnant women who do not exercise and were not active before pregnancy.

In our study, we investigated the effects of a single bout of maximal physical exertion and an 8-week, online-monitored HIIT intervention on parameters related to PPT, PTOL, and subjective pain perception. To our knowledge, this is the first study to examine the effects of maximal progressive exercise testing and HIIT on pain sensitivity in pregnant women as measured by pain threshold and pain tolerance. Most studies have focused on changes in a subjective perception of persistent pain, using only self-reported data from women who participated in traditional childbirth classes. Although there are some studies in which authors have examined pain perception in response to actual pain stimuli such as pressure pain (a mechanical stimulus) or the Cold Pressor Test (CPT) (Ohel et al., 2007; Parent Vachon et al., 2019), most of them have focused mainly on analyzing specific factors that affect only postpartum pain (Berlit et al., 2018). Thus, taking into account the subjective aspect of pain and the specificity of the physiological and psychological processes of a pregnant woman, our experimental pain measurements emphasize a common interpretation of the sensory phenomenon of pain. Our main finding in this study was that pregnant women share the same mechanisms of exercise-induced pain sensitivity as men, athletes, the elderly, and nonpregnant women. Previously published research has confirmed that repetitive exposure to a high-intensity exercise stimulus increases muscle pain tolerance. HIIT confirmed the effects of high-intensity training on pain tolerance (O’Leary et al., 2017) and pain threshold (Mijwel et al., 2018).

In our study, we did not observe any significant effects of HIIT intervention and CPET on pain threshold in pregnant women, which is consistent with previously published studies with different groups of participants that are inconclusive for this trait (Koltyn, 2000; Koltyn, 2002). However, our participants achieved significantly higher pain tolerance after 8 weeks of HIIT, a finding consistent with the effects of similar interventions in other groups (Tesarz et al., 2012). In addition, we observed the occurrence of acute exercise-induced hypoalgesia, as pain tolerance was significantly higher after CPET than before CPET.

From the result of our study, it can be inferred that high-intensity physical activity is positively associated with improved pain modulation. Specifically, pregnant women who participated in HIIT training showed increased pain tolerance. Interestingly, in addition to the positive effect of HIIT on pain tolerance, we also found a significant and positive effect of such an intervention on subjective pain perception. It may be seen as counterintuitive, as it seems that HIIT increased the subjective experience of pain. However, we believe this means that study participants were able to endure objectively stronger pain stimuli while being aware of its high subjective intensity. This phenomenon may potentially lead to increased pain tolerance during labor and puerperium, and could contribute significantly to the overall wellbeing and comfort of pregnant women during labor as well as allow for more effective breastfeeding. However, further research in this area is needed to confirm this hypothesis.

Apart from the relatively small sample, one of the weaknesses of this study is that we collected the data only twice in a relatively short period of time—before and after an 8-week training program. It would be valuable to evaluate potential analgesia related to physical training at different time points during pregnancy and postpartum. In addition, this study lacks a comparison group of women who were either inactive during pregnancy or who participated in exercise programs of an intensity other than HIIT, such as the far more common moderate - intensity training. Another interesting topic not addressed in this study is the analysis of changes in pain perception in women with multiple or complicated pregnancies who participated in appropriately adapted PA programs. Such data could support the development of strategies that promote both healthy lifestyles and pain management in a much larger group of women.

## 5 Conclusion

In conclusion, our results describe for the first time that post-exercise analgesia may be important in pregnant women. Increased pain tolerance before labor could lead to better management of pain during labor and in the postpartum and lactation periods. Increasing pregnant women’s awareness of this issue can improve their wellbeing and provide more comfort during labor. We can also conclude that HIIT intervention seems to be a very beneficial exercise method for pregnant women to improve their pain tolerance while being obstetrically safe. It should therefore be widely promoted to women before and during pregnancy, as well as to sports and health professionals who promote and support women’s participation in exercise programs.

## Data Availability

The raw data supporting the conclusion of this article will be made available by the authors, without undue reservation.
